# Perioperative regional anaesthesia and postoperative longer-term outcomes

**DOI:** 10.12688/f1000research.9100.1

**Published:** 2016-10-11

**Authors:** Jan Jakobsson, Mark Z. Johnson

**Affiliations:** 1 Department of Anaesthesia & Intensive Care, Institution for Clinical Science, Karolinska Institutet, Danderyds University Hospital, Stockholm, Sweden; 2 Department of Anaesthesia & Critical Care, Mater Misercordiae University Hospital, Dublin, Ireland; 3 School of Medicine, University College Dublin, Dublin, Ireland

**Keywords:** local anaesthesia, neuraxial block, inflammation, immune function, cancer re-occurrence, metastasis

## Abstract

Regional anaesthesia provides effective anaesthesia and analgesia in the perioperative setting. Central neuraxial blocks—that is, spinal and epidural blocks—are well established as an alternative or adjunct to general anaesthesia. Peripheral blocks may be used as part of multimodal anaesthesia/analgesia in perioperative practice, reducing the need for opioid analgesics and enhancing early recovery. Furthermore, regional anaesthesia has increased in popularity and may be done with improved ease and safety with the introduction of ultrasound-guided techniques. The effects of local anaesthetics and regional anaesthesia on long-term outcomes such as morbidity, mortality, the quality of recovery beyond the duration of analgesia, and whether it can expedite the resumption of activities of daily living are less clear. It has also been suggested that regional anaesthesia may impact the risk of metastasis after cancer surgery. This article provides an overview of current evidence around quality of recovery, risk for delirium, long-term effects, and possible impact on cancer disease progression associated with the clinical use of local and regional anaesthetic techniques. In summary, there is still a lack of robust data that regional anaesthesia has a clinical impact beyond its well-acknowledged beneficial effects of reducing pain, reduced opioid consumption, and improved quality of early recovery. Further high-quality prospective studies on long-term outcomes are warranted.

## Introduction

The effect of local anaesthesia on nerve conductivity has a long history. Modern amide local anaesthetics are reassuringly safe, providing dose-dependent, reversible nerve conduction blockade. The beneficial effects of regional anaesthesia on postoperative pain are well known. Local anaesthesia may be administered by local infiltration, peripheral nerve block, or central neuraxial block depending on what effects are sought. Local infiltration anaesthesia is simple and safe. Peripheral nerve blocks are increasingly popular because of ultrasound techniques, which provide enhanced visualisation of its performance. The positive effects on postoperative pain and reduced opioid consumption are well acknowledged. There are recent reviews of upper-extremity blocks ^1^, lower-extremity blocks ^2^, and abdominal-wall blocks ^3^ that demonstrate these effects. There may not be a major difference between local infiltration and peripheral nerve blocks for the management of postoperative pain in the first 24 hours following some surgical procedures (for example, total hip replacement ^4^) in contrast to total knee arthroplasty (TKA), where wide variability in postoperative pain scores is documented. In a Cochrane meta-analysis, Chan *et al*. supported the use of regional anaesthesia for postoperative analgesia following TKA ^5^. In May 2016, Hu *et al*. published a meta-analysis showing that the two techniques were similarly effective for early pain management following TKA ^6^.

There is also increasing interest in the systemic effects of local anaesthetics. The potential beneficial effect of local anaesthesia on inflammation and immune function has long been suggested. In 2000, Hollmann and Durieux ^7^ had already addressed the potential anti-inflammatory effects of local anaesthetics, reviewing the literature on local anaesthetic effects on the inflammatory response and especially on inflammatory cells (mainly polymorphonuclear granulocytes but also macrophages and monocytes) ^8^. The exact mechanisms of action are not clear but seem to involve a reversible interaction with membrane proteins and lipids, thus regulating cell metabolic activity, migration, exocytosis, and phagocytosis ^9, 10^. The clinical anti-inflammatory effect of intravenous lidocaine is today reasonably well documented. The beneficial effects of intravenous lidocaine on recovery have been described; this technique has been shown to reduce the time to return of bowel function and shorter hospital stay after abdominal surgery ^11^. The most recent meta-analysis, published in 2016 ^12^, supports the beneficial effects on pain but calls for further studies, as the quality of evidence was limited as a result of trial heterogeneity and small sample sizes. There are also several studies looking at the potential effect of local anaesthetics on immune function ^13– 15^. The findings of these studies have not necessarily been shown to have clinical significance and have yet to be translated into routine clinical practice.

## Central blocks, epidural and spinal anaesthesia, and morbidity and mortality in general surgery

Neuraxial blocks are widely used in addition or as an alternative to general anaesthesia for general surgery as well as for postoperative pain management. The beneficial effects during the early phase of recovery are well documented. A recent meta-analysis comparing general anaesthetic with epidural and general anaesthetic with opioid analgesia showed improved pain control and bowel function in the epidural and general anaesthesia group ^16^. The impact of regional anaesthesia on longer-term outcomes has become of increasing interest ^17^. An overview of Cochrane systematic reviews of neuraxial blockade for the prevention of postoperative mortality and major morbidity found weak evidence for reduced mortality and pneumonia in high-risk patients who received neuraxial anaesthesia ^18^. There is still a lack of evidence to show a difference in morbidity and mortality between regional anaesthesia and general anaesthesia for urological and vascular surgery ^19, 20^. In 2014, Popping *et al*. published a meta-analysis supporting the use of combined general and epidural anaesthesia, since epidural analgesia reduced mortality and improved a multitude of cardiovascular, respiratory, and gastrointestinal morbidity endpoints compared with patients receiving systemic analgesia ^21^. However, this article had numerous limitations, including certain methodological flaws and the inclusion of unpublished data.

## Regional anaesthesia and orthopaedic surgery

In a meta-analysis published in 2013, Barbosa *et al*. found no significant long-term benefit associated with regional anaesthesia as compared with general anaesthesia for orthopaedic surgery ^22^. Memtsoudis *et al*. found that regional anaesthesia reduced the risk of major complications in a subset of patients with obstructive sleep apnoea undergoing joint arthroplasty in comparison with patients who received combined neuraxial and general or general anaesthesia alone (16.0%, 17.2%, and 18.1%, respectively; *P* = 0.0177) ^23^.

However, the benefits associated with epidural anaesthesia have been questioned. The quality of studies included in meta-analyses is low and some of the positive effects are merely dependent on single studies in high-risk patients ^24^. The potential benefit on morbidity and mortality from regional anaesthesia for acute femoral fracture repair surgery has also recently been challenged. Neuman *et al*. showed shorter hospital stay but no difference in mortality ^25^. Seitz *et al*. showed regional anaesthesia to be associated with similar mortality but a lower need for intensive care unit admission compared with general anaesthesia in older adults with dementia who underwent surgery for hip fracture repair ^26^. Brox *et al*. found one-year mortality to be only marginally lower in patients with neuraxial anaesthesia compared with general anaesthesia (odds ratio (OR) = 0.84, confidence interval (CI) 0.70–1.0) but to be similar in patients with combined regional and general anaesthesia (OR = 1.3, CI 0.70–2.3) ^27^. White *et al*. likewise found no difference in mortality associated with neuraxial block or general anaesthesia but emphasised the importance of maintaining adequate blood pressure ^28^. In February 2016, Johnson *et al*. published a meta-analysis of neuraxial anaesthesia compared with general anaesthesia for hip or knee arthroplasty (or both) showing no significant differences in any outcomes studied: mortality, surgical duration, surgical site or chest infections, nerve palsies, postoperative nausea and vomiting, or thromboembolic disease when anti-thrombotic prophylaxis was used ^29^.

Thus, regional anaesthesia provides effective early postoperative analgesia and is a valid alternative to general anaesthesia. The effects on early postoperative pain are well documented, but its impact beyond the early period and effects on major morbidity and mortality are not obvious.

## Regional anaesthesia and delirium

Postoperative delirium (PD) is commonly seen in the elderly. The risk in the elderly having orthopaedic surgery has been reported to be around 17% with huge variability ^30^. The impact of anaesthetic technique on early PD and more long-lasting cognitive deficits such as postoperative cognitive dysfunction (POCD) has been under scrutiny for decades. In 1995, Williams-Russo *et al*. ^31^ compared epidural and general anaesthesia in 262 patients undergoing elective primary total knee replacement with a median age of 69 years. The type of anaesthesia—general or neuraxial—did not affect the magnitude or pattern of POCD. The International Study of Postoperative Cognitive Dysfunction (ISPOCD) group ^32^ published the results of a multi-centre study assessing POCD and the impact of anaesthetic technique, general anaesthesia versus regional anaesthesia. Early testing (one week postoperatively) showed a higher incidence among patients having general anaesthesia, but no difference was noted at 3 months. In 2010, Mason *et al*. conducted a meta-analysis and found no impact of anaesthetic technique on the risk for PD, but general anaesthesia was found to have a small, non-significant association with an increased rate of POCD (OR = 1.34, CI 0.93–1.95) ^33^. There was no evidence of publication bias. Davis *et al*. came to a similar conclusion in a meta-analysis published in 2014; of 16 included studies, 13 showed no difference and three showed some minor effect ^34^. There is a need for further high-quality studies, and there are ongoing studies on this topic.

## Regional anaesthesia and quality of recovery

The impact of regional anaesthesia on the quality of recovery, as assessed by objective multi-dimensional tools (for example, Quality of Recovery 15 or Postoperative Quality of Recovery score), beyond the early postoperative phase is sparsely studied. Catro-Alves *et al*. compared spinal anaesthesia and general anaesthesia for elective hysterectomy and assessed recovery by using the global quality of recovery-40 questionnaire (QoR-40) up to 48 hours after surgery but not beyond ^35^. The beneficial effects on pain and quality of early recovery were confirmed. Liu *et al*. conducted a study amongst elderly patients undergoing knee arthroplasty in general anaesthesia or peripheral block and followed intraoperative and postoperative course up to day 7 with the Postoperative Quality of Recovery Scale (PostopQRS) tool ^36^. The intraoperative and early postoperative course favoured the block technique, but at day 7 no difference was found. Resumption of activities of daily living and patient satisfaction were similar between the two groups.

## Regional anaesthesia and cancer

There is considerable interest in whether local anaesthetics and regional anaesthesia may impact cancer progression and recurrence. The potential mechanisms by which cancer progression may be influenced include attenuation of the surgical stress response, reduced pain and opioid requirements, and also the direct action that some local anaesthetics may have on cancer cells. It is also suggested that local anaesthetics and regional anaesthesia may have an effect on immune function and inflammation. There are studies assessing immune function in the experimental setting ^37^. Despite mounting *in vitro* data suggesting positive effects ^38, 39^, *in vivo* studies and well-designed human trials are lacking ^40^. The clinical relevance of these results must be interpreted with great caution and cannot necessarily be directly translated into human clinical practice. Several recent articles have raised the question of whether anaesthetic technique and drug choice could influence long-term tumour progression ^41, 42^. However, these articles are retrospective analyses of studies designed to address a different clinical question. In 2006, Exadaktylos *et al*. published a retrospective study suggesting that paravertebral block in combination with general anaesthesia for primary breast cancer surgery could reduce the risk for tumour progression as compared with standard anaesthesia and postoperative opioids for pain management ^43^. In 2014, however, a Cochrane systematic review concluded that the evidence is insufficient to make any firm recommendations ^44^ *.*

Although regional anaesthesia provides effective early postoperative analgesia and promotes enhanced recovery, there is insufficient data to promote a change in clinical practice in order to affect cancer progression ^45– 48^. We await results from ongoing studies to better define the role of anaesthesia for patients with cancer ^49^.

## Conclusions

Regional anaesthesia is effective at reducing pain and opioid consumption during the early postoperative phase. The impact of regional anaesthesia on the risk for cognitive side effects of surgery, PD, and POCD seems minor. Data on the effects of local anaesthesia, local infiltration, and regional anaesthesia on quality of recovery assessed by multi-domain tools are sparse. The documentation around long-term outcomes and the potential beneficial effects on morbidity and mortality is also weak, and further high-quality studies are warranted. The potential effect on cancer progression is not proven. There are several ongoing prospective randomised studies that may help define whether regional anaesthesia techniques could not only provide effective postoperative pain but also impact cancer progression. However, it should be acknowledged that the number of patients needed to have sufficient power to show any potential significant effect is large, such studies are expensive, and long follow-up times are required.
